# TAB3 upregulates Survivin expression to promote colorectal cancer invasion and metastasis by binding to the TAK1-TRAF6 complex

**DOI:** 10.18632/oncotarget.22497

**Published:** 2017-11-18

**Authors:** Chen Luo, Rongfa Yuan, Leifeng Chen, Wei Zhou, Wei Shen, Yumin Qiu, Jun Shao, Jinlong Yan, Jianghua Shao

**Affiliations:** ^1^ Department of General Surgery, Second Affiliated Hospital of Nanchang University, Nanchang 330006, China; ^2^ Jiangxi Province Key Laboratory of Molecular Medicine, Nanchang 330006, China; ^3^ Department of Gastrointestinal Surgery, Jiangxi Provincial Cancer Hospital, Nanchang 330029, China

**Keywords:** TAB3, Survivin, NF-κB pathway, colorectal cancer, metastasis

## Abstract

Transforming growth factor-β-activated kinase 1 (TAK1)-binding protein 3 (TAB3) is involved in cancer proliferation and metastasis, but its role in colorectal cancer remains unclear. In this study, we demonstrated that TAB3 is upregulated in colorectal cancer tissues and that high TAB3 levels correlated with tumor metastasis and a poor prognosis in colorectal cancer. In addition, TAB3 knockdown decreased Survivin expression and suppressed colorectal cancer cell migration and invasion *in vitro*, and reduced liver metastasis *in vivo*. Importantly, we found that TAB3 regulated Survivin expression by activating the NF-κB pathway through the formation of the TAK1-TAB3-TRAF6 complex. These findings suggest TAB3 may be a useful prognostic biomarker in colorectal cancer and a target for treatment of metastatic colorectal cancer.

## INTRODUCTION

Colorectal cancer (CRC) is one of the most common types of cancer and a leading cause of cancer-related deaths worldwide [1]. Metastasis and recurrence are the major causes of death in CRC. Although surgical resection is regarded as the standard curative treatment for CRC, the metastasis and recurrence rates after radical resection of CRC remain high [2]. Therefore, it is urgent to reveal the underlying molecular mechanisms involved in CRC progression and metastasis.

Survivin, a member of the inhibitor of apoptosis (IAP) protein family that inhibits caspases and blocks cell death, is highly expressed in most cancers and is associated with a poor clinical outcome [3, 4]. Overexpression of Survivin has been reported in almost all human malignancies, including bladder cancer, lung cancer, breast cancer, stomach cancer, esophageal cancer, liver cancer, ovarian cancer and other cancer tissues [4–6]. Recently, emerging evidence has linked Survivin to tumor metastasis. For example, prior studies demonstrated that knockdown of Survivin in glioma inhibited angiogenesis [7], Survivin overexpression enhanced human melanocyte and melanoma cell migration [8] and Survivin promoted tumor cell invasion *in vitro* and metastatic dissemination in an *in vivo* murine model of breast cancer [9]. In CRC, Survivin overexpression is an independent poor prognostic factor in patients, and knockdown of Survivin could significantly inhibit CRC invasion and metastasis [10].These studies suggest that Survivin may play an important role in tumor invasion and metastasis.

In addition, previous studies have shown that Survivin is regulated by the NF-κB and PI3K/AKT signaling pathways in many cancers, such as bladder cancer, lymphoma and pancreatic cancer [11–14]. However, the mechanism by which Survivin is regulated in the process of CRC invasion and metastasis remains unclear.

Transforming growth factor-β-activated kinase 1 (TAK1) is a member of the mitogen-activated protein kinase kinase kinase (MAPKKK) family, which is involved in signal transduction, inflammation and innate immune responses [15–17]. It is well established that TAK1 forms a complex with its binding proteins (TAB1, TAB2, and TAB3) and the TNF-receptor-associated factors (TRAFs), whereupon it stimulates NF-κB and mediates a wide range of biological processes [18, 19]. TAB3, as a newly identified TAK1 binding partner, has been implicated in the immune response, signal transduction, inflammation and autophagy [20–22]. Recently, there has been an increasing number of studies on TAB3 in malignant tumors. TAB3 is markedly overexpressed in various tumor tissues, such as the testis, skin, non-small cell lung cancer (NSCLC), hepatocellular carcinoma (HCC) and small intestinal cancers [23–25]. In addition, a previous study reported that silencing of TAB3 inhibits NSCLC proliferation and chemoresistance via the NF-κB pathway [24]. In HCC, knockdown of TAB3 has been shown to enhance the rate of doxorubicin-induced apoptosis and chemosensitivity of HCC cells by acting through the NF-κB pathway [26]. Also notably, TAB3 O-GlcNAcylation has been shown to promote breast cancer metastasis [27]. However, the role and specific mechanism of TAB3 in the process of CRC invasion and metastasis remain unclear.

In this study, we first demonstrated that TAB3 is upregulated in CRC tissues compared with non-tumor tissues and that the overexpression of TAB3 is associated with metastasis and poor survival of CRC patients. Furthermore, functional studies provided the first evidence that TAB3 facilitates CRC invasion *in vitro* and metastasis *in vivo* by upregulating Survivin expression. We also demonstrated that TAB3 regulates Survivin through the NF-κB signaling pathway and activates NF-κB through binding to the TAK1-TRAF6 complex.

## RESULTS

### High TAB3 expression is associated with metastasis and poor overall survival in CRC

**Figure 1:**
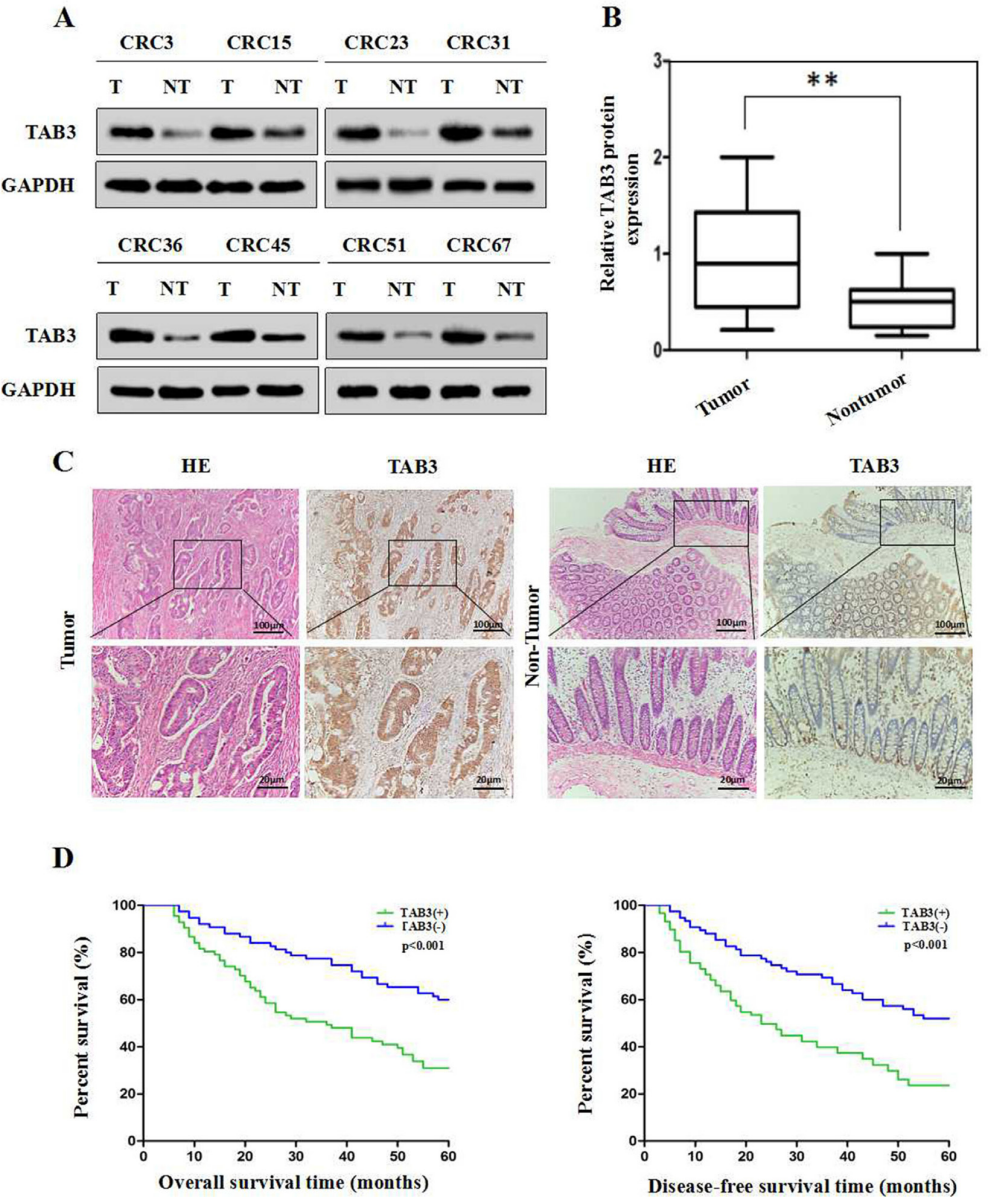
High TAB3 expression is associated with a poor prognosis in CRC patients (**A**) Representative Western blot analysis of TAB3 protein expression (T: tumor, NT: non-tumor tissues). (**B**) Quantification of TAB3 protein expression using Western blot analyses in 68 paired CRC and their adjacent non-tumor tissues. GAPDH protein expression was used as an internal control (^**^P < 0.01). (**C**) IHC showed that TAB3 protein levels were increased in CRC tissues (magnification: 100×, inset magnification: 400×). (**D**) Kaplan-Meier survival curves for 162 CRC patients. Patients with high TAB3 protein expression levels showed significantly poorer overall survival and disease-free survival than patients with low TAB3 protein expression (both *P* < 0.001, log-rank test).

To explore the expression and clinical significance of TAB3 in CRC, we first examined TAB3 expression in 68 CRC tissue samples and corresponding adjacent normal tissues using western blotting. The western blotting results showed that TAB3 protein was markedly higher in 58.82% (40/68) of the CRC tissue samples than in their adjacent normal tissue (Figure 1A and 1B). We next examined TAB3 expression in 162 CRC tissue samples and their paired adjacent normal tissues by immunohistochemistry (IHC). The IHC results showed that the TAB3 protein was highly expressed in 53.70% (87/162) of the CRC tissue samples and in only 8.64% (14/162) of the adjacent tissues (Figure 1C). These findings strongly indicate that TAB3 is overexpressed in CRC.

Next, we analyzed the association between TAB3 expression and clinicopathological parameters. The results revealed that high TAB3 expression was significantly associated with lymphatic metastasis (*P <* 0.001), venous invasion (*P =* 0.036) and advanced TNM stage (*P =* 0.001), indicating that TAB3 overexpression is involved in CRC aggressiveness and metastasis (Table 1). Furthermore, CRC patients in the high TAB3 expression group had a much shorter median survival time than those in the low TAB3 expression group (Figure 1D). Moreover, univariate and multivariate analyses using the Cox regression model revealed that the TAB3 level, lymphatic metastasis, venous invasion and TNM classification could be recognized as independent prognostic factors to evaluate the outcome of CRC patients (Supplementary Tables 1 and 2). This suggests that TAB3 potentially has clinical value as a predictive biomarker for disease outcome in CRC.

**Table 1:** Relationship between TAB3 expression and clinicopathological features in 162 CRC patients

Parameters	Case	TAB3 expression		*P* value
	162	Low	High	
Age (years)				
<60	102	51	51	0.255
≥60	60	24	36	
Gender				
Female	77	40	37	0.207
Male	85	35	50	
Tumor size (cm)				
<5	88	46	42	0.115
≥5	74	29	45	
Histological type				
Differentiated	130	65	65	0.075
Undifferentiated	32	10	22	
Lymphatic metastasis				
Negative	91	58	33	<0.001^**^
Positive	71	17	54	
Venous invasion				
Negative	116	60	56	0.036^*^
Positive	46	15	31	
TNM staging				
I/II	97	55	42	0.001^**^
III/IV	65	20	45	

*P* value was generated by comparing all subgroups and analyzed by the chi-square test. *P* < 0.05 was considered as statistically significant.

### Downregulation of TAB3 reduces Survivin expression and represses CRC invasion and metastasis *in vitro* and *in vivo*

**Figure 2:**
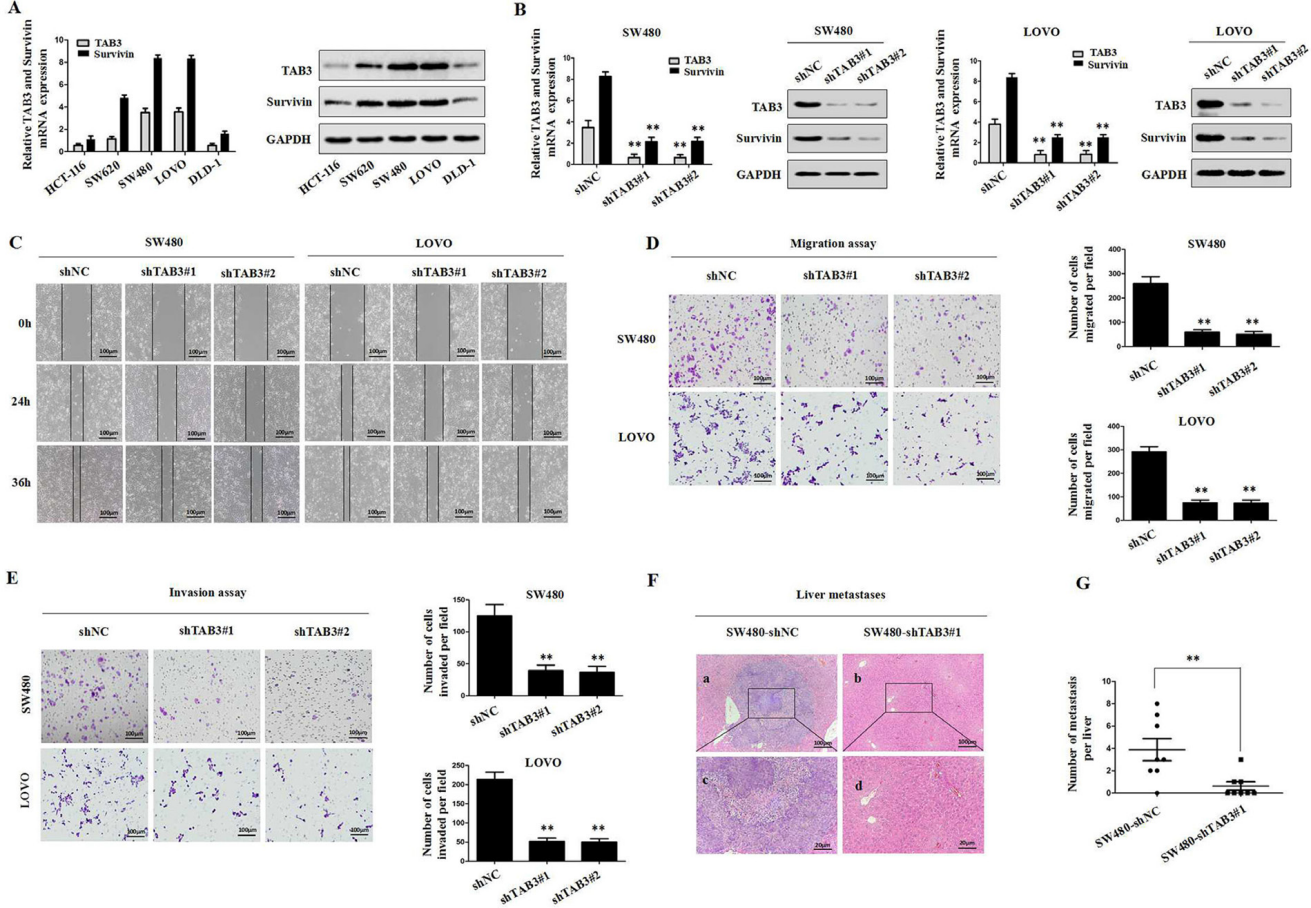
Stable knockdown of TAB3 reduced Survivin expression and inhibited CRC invasion and metastasis *in vitro* and *in vivo* (**A**) qRT-PCR and Western blot analyses of TAB3 and Survivin expression in the indicated CRC cell lines. (**B**) Western blot analyses were used to detect TAB3 and Survivin expression in SW480 and LOVO cells stably transfected with shNC, shTAB3#1 and shTAB3#2. (**C**) Wound healing assay. Wound closure was delayed in stable TAB3-knockdown cells compared with the shNC control at both the 24- and 36-h time points. Magnification, ×100. (**D**) Transwell migration assays of SW480 and LOVO cells with TAB3 expression stably inhibited (^**^p < 0.01). Magnification, ×100. (**E**) Transwell invasion assays of SW480 and LOVO cells treated with shNC, shTAB3#1 and shTAB3#2. Magnification, ×100. (**F**) Representative H&E staining of livers from the SW480-shNC and SW480-shTAB3 #1 groups. Magnification: a and b, ×100; c and d, ×400. (**G**) (*n* = 8; ^**^P < 0.01).

To investigate whether TAB3 regulated Survivin expression in CRC cells, we first examined the levels of TAB3 and Survivin in various CRC cells by qRT-PCR and immunoblotting assays. Our results indicated that TAB3 was positively correlated with Survivin (Figure 2A). We then stably transfected the TAB3-specific short hairpin RNA (shTAB3#1 and shTAB3#2) into SW480 and LOVO cells to knockdown TAB3. qRT-PCR and western blotting results showed that the knockdown of TAB3 could decrease Survivin expression in the SW480 and LOVO cells (*P <* 0.01, Figure 2B). Next, we observed that cell migration was significantly decreased in the shTAB3#1 and shTAB3#2 groups (*P <* 0.01, Figure 2C and 2D). Using a Matrigel-coated Transwell chamber, we found that the stable TAB3 knockdown SW480 and LOVO cells invaded through the matrix slower than that in the control group (*P <* 0.01, Figure 2E). Furthermore, an *in vivo* metastasis experiment further confirmed that the tumors formed by SW480-shTAB3#1 cells showed decreased liver metastasis compared with the tumors formed by control cells (Figure 2F and 2G).

To verify the specificity of this tumor-promoting effect, we developed stable clones with TAB3 overexpression from HCT-116 and DLD-1 cells. The results showed that the overexpression of TAB3 led to marked upregulation of Survivin (Supplementary Figure 1A). The overexpression of TAB3 strongly enhanced *in vitro* CRC cell migration and invasion (Supplementary Figure 1B–1D). Consistent with the *in vitro* results, TAB3 overexpression increased liver metastasis in nude mice compared to control groups (Supplementary Figure 1E and 1F). Taken together, the results showed that the stable knockdown of TAB3 reduces Survivin expression and inhibit CRC invasion and metastasis.

### Survivin is required for TAB3-mediated CRC invasion and metastasis *in vitro* and *in vivo*

**Figure 3:**
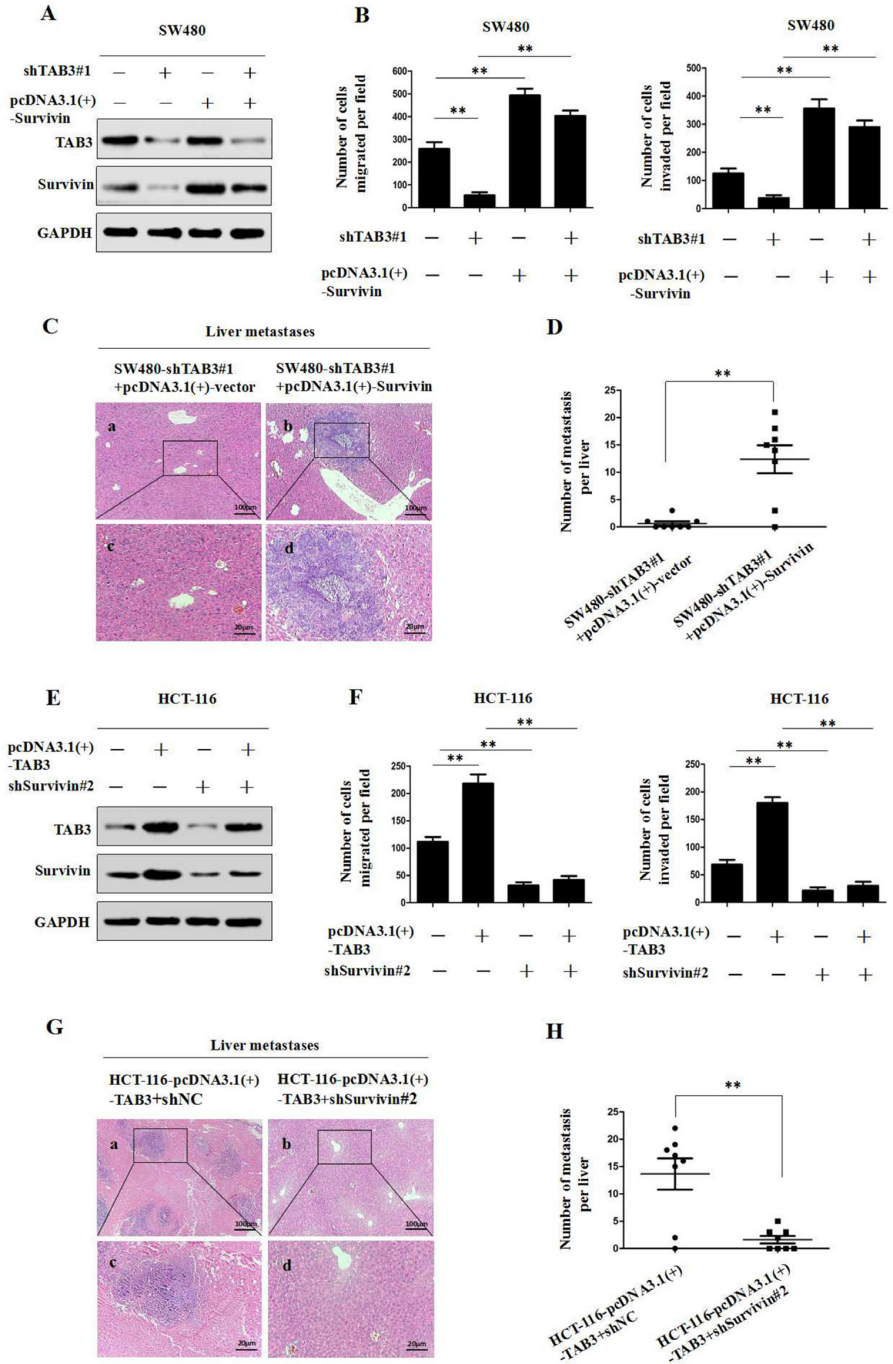
TAB3 promotes CRC invasion and metastasis by upregulating Survivin expression (**A**) The upregulation of Survivin attenuated the loss of Survivin expression in SW480-shTAB3 cells. (**B**) Transwell assays showed that the upregulation of Survivin significantly rescued cell migration and invasion in SW480-shTAB3 cells (^**^P < 0.01). (**C**) Representative H&E staining of livers from the SW480-shTAB3#1 +pcDNA3.1(+)-vector and SW480-shTAB3#1 +pcDNA3.1(+)-Survivin groups is shown. Magnification: a and b, ×100; c and d, ×400. (**D**) (*n* = 8; ^**^P < 0.01). (**E**) Protein levels of TAB3 and Survivin were detected by Western blot analysis. The knockdown of Survivin expression dramatically inhibited the increase of Survivin expression in HCT116-TAB3 cells. (**F**) Transwell assays showed that Survivin inhibition reduced TAB3-enhanced cell migration and invasion (^**^P < 0.01). (**G**) Representative H&E staining of livers from the HCT116-TAB3+shNC and HCT116-TAB3+shSurvivin#2 groups is shown. Magnification: a and b, ×100; c and d, ×400. (**H**) (*n* = 8; ^**^P < 0.01).

We utilized western blot and transwell assays to further investigate TAB3’s regulation of Survivin and influence on CRC invasion and metastasis. The immunoblot analysis results showed that the knockdown of TAB3 decreased Survivin expression (Figure 3A and Supplementary Figure 2A), whereas the upregulation of Survivin attenuated the loss of Survivin expression in TAB3 knockdown SW480 and LOVO cells. We also found that the downregulation of TAB3 dramatically decreased the migration and invasion of SW480 and LOVO cells, whereas the upregulation of Survivin rescued the decreased migration and invasion abilities induced by TAB3 knockdown (Figure 3B and Supplementary Figure 2B). Furthermore, the *in vivo* assay also showed that the upregulation of Survivin rescued the decreased incidence of liver metastasis of the SW480-shTAB3#1 group (Figure 3C and 3D).

Next, we decreased Survivin expression in TAB3-overexpressing CRC cells and then measured TAB3 and Survivin protein levels and cell migration and invasion. Immunoblotting showed that the overexpression of TAB3 significantly increased Survivin expression, whereas Survivin knockdown dramatically inhibited the increase in Survivin expression induced by TAB3 in HCT-116 and DLD-1 cells (Figure 3E and Supplementary Figure 2C). Meanwhile, the downregulation of Survivin significantly reduced TAB3-enhanced cell migration and invasion (Figure 3F and Supplementary Figure 2D). *In vivo* results showed that downregulation of Survivin decreased the incidence of liver metastasis of the HCT-116-pcDNA3.1(+)-TAB3 group (Figure 3G and 3H). Thus, these results demonstrate that Survivin is essential for TAB3-mediated CRC metastasis.

### TAB3 regulates Survivin expression through the NF-κB pathway in CRC cells

**Figure 4:**
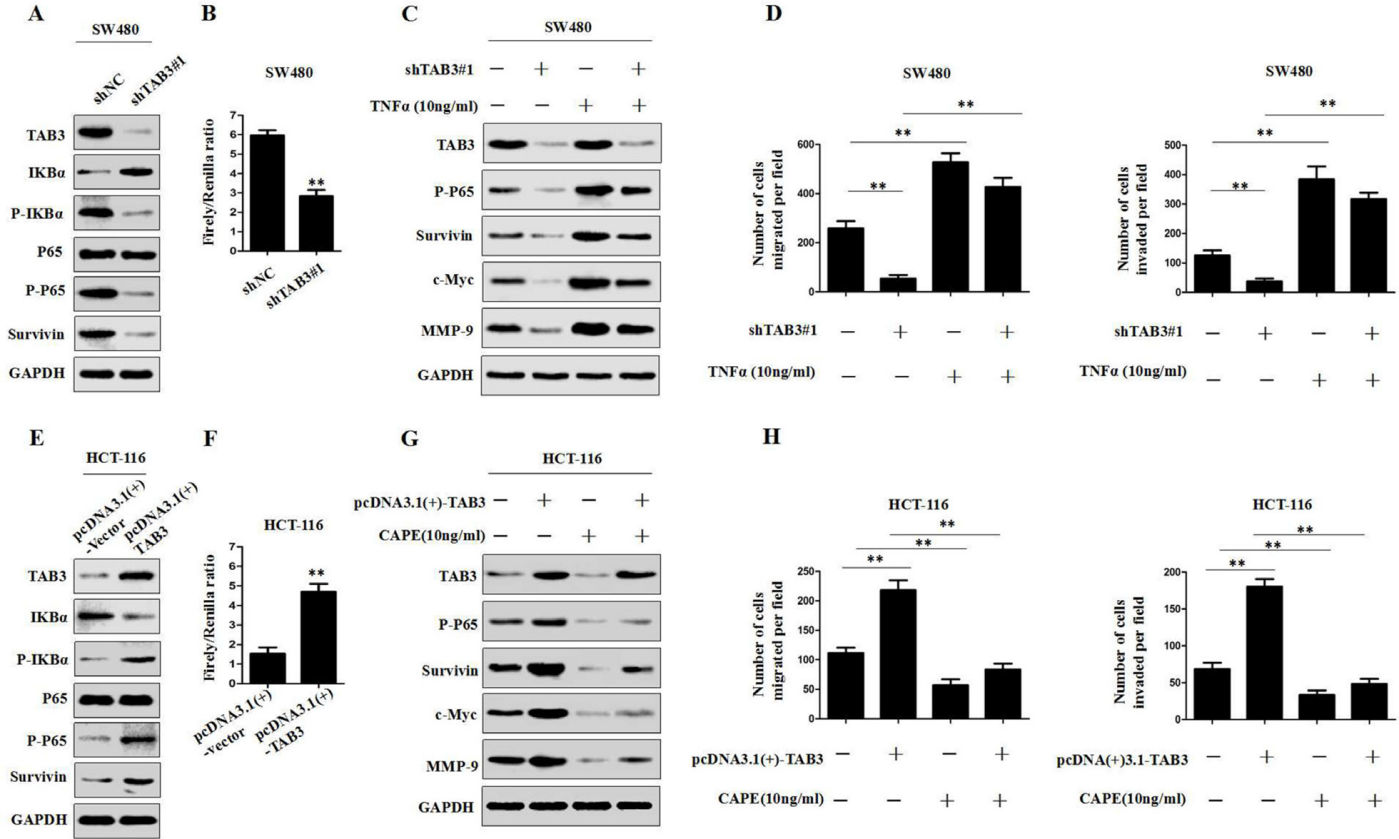
TAB3 regulates Survivin expression through the NF-κB pathway (**A**) The protein expression level of TAB3, IKBa, p-IKBa, P65, p-P65 and Survivin were assessed by Western blotting in TAB3-silenced SW480 cells. (**B**) Luciferase analysis was used to determine the NF-κB activity in TAB3-knockdown SW480 cells ( ^**^P < 0.01). (**C**) Western blot analysis showing the levels of TAB3 silencing and activation of the NF-κB pathway (treatment with 10 ng/ml tumor necrosis factor) and their effects on p-P65, Survivin, c-Myc, and MMP-9 in SW480 cells. (**D**) Activation of the NF-κB pathway rescued cell migration and invasion in SW480-shTAB3 cells (^**^P < 0.01). (**E**) Protein expression levels of TAB3, IKBa, p-IKBa, P65, p-P65 and Survivin in HCT116 cells transfected with pcDNA3.1(+)-vector and pcDNA3.1(+)-TAB3. (**F**) Luciferase analysis was performed using HCT116 cells transfected with the pcDNA3.1(+)-vector or pcDNA3.1(+)-TAB3 plasmid (^**^P < 0.01). (**G**) Western blot analysis showing the levels of TAB3 overexpression and NF-κB signaling inhibition (treatment with 10 ng/ml Caffeic Acid Phenethyl Ester) and their effects on p-P65, Survivin, c-Myc and MMP-9 in HCT116 cells.(**H**.) Blockade of the NF-κB pathway enhanced migration and invasion in HCT116-pcDNA3.1(+)-TAB3 cells (^**^P < 0.01).

To further clarify the mechanism by which TAB3 regulates Survivin in CRC cells, we hypothesized that TAB3 regulates Survivin via the NF-κB signaling in CRC cells. We first measured the changes in phosphorylated-IκBα (p-IκBα), phosphorylated-p65 (p-p65), total p65 expression and NF-κB activity in TAB3 knockdown SW480 and LOVO cells. The results showed that the knockdown of TAB3 can significantly decrease p-IκBα, p-p65 and the activity of NF-κB luciferase reporter in SW480 and LOVO cells (Figure 4A, 4B and Supplementary Figure 3A, 3B). We also found that the knockdown of TAB3 decreased other downstream genes in the NF-κB pathway, including c-Myc and MMP-9 in SW480 and LOVO cells. Whereas, the activation of NF-κB signaling by treatment with TNFα (10 ng/ml) attenuated the loss of this protein expression in TAB3-knockdown SW480 and LOVO cells (Figure 4C and Supplementary Figure 3C). In addition, we found that the activation of NF-κB signaling rescued the decreased cell migration and invasion induced by the knockdown of TAB3 (Figure 4D and Supplementary Figure 3D).

To further verify that TAB3 regulates Survivin expression through the NF-κB pathway, we investigated the effect of TAB3 overexpression on the NF-κB pathway. The results showed that the upregulation of TAB3 expression significantly increased p-IκBα, p-p65 and the activity of NF-κB luciferase reporter (Figure 4E–4F and Supplementary Figure 3E–3F). We then detected the expression of Survivin, c-Myc and MMP-9 in HCT-116- and DLD-1-pcDNA3.1(+)-TAB3 cells treated with the NF-κB inhibitor caffeic acid phenethyl ester (CAPE, 10 ng/ml). The results showed that the overexpression of TAB3 increased the expression of Survivin, c-Myc and MMP-9 proteins. Whereas, blockade of NF-κB signaling dramatically inhibited the increase of Survivin, c-Myc and MMP-9 expression in HCT-116- and DLD-1-pcDNA3.1(+)-TAB3 cells (Figure 4G and Supplementary Figure 3G). Meanwhile, the transwell assay showed that blockade of NF-κB signaling dramatically decreased TAB3-induced cell migration and invasion (Figure 4H and Supplementary Figure 4H). These studies confirmed that TAB3 regulates Survivin-induced CRC migration and invasion through the NF-κB pathway.

### TAB3 activates NF-κB by binding to the TAK1-TRAF6 complex in CRC cells

**Figure 5:**
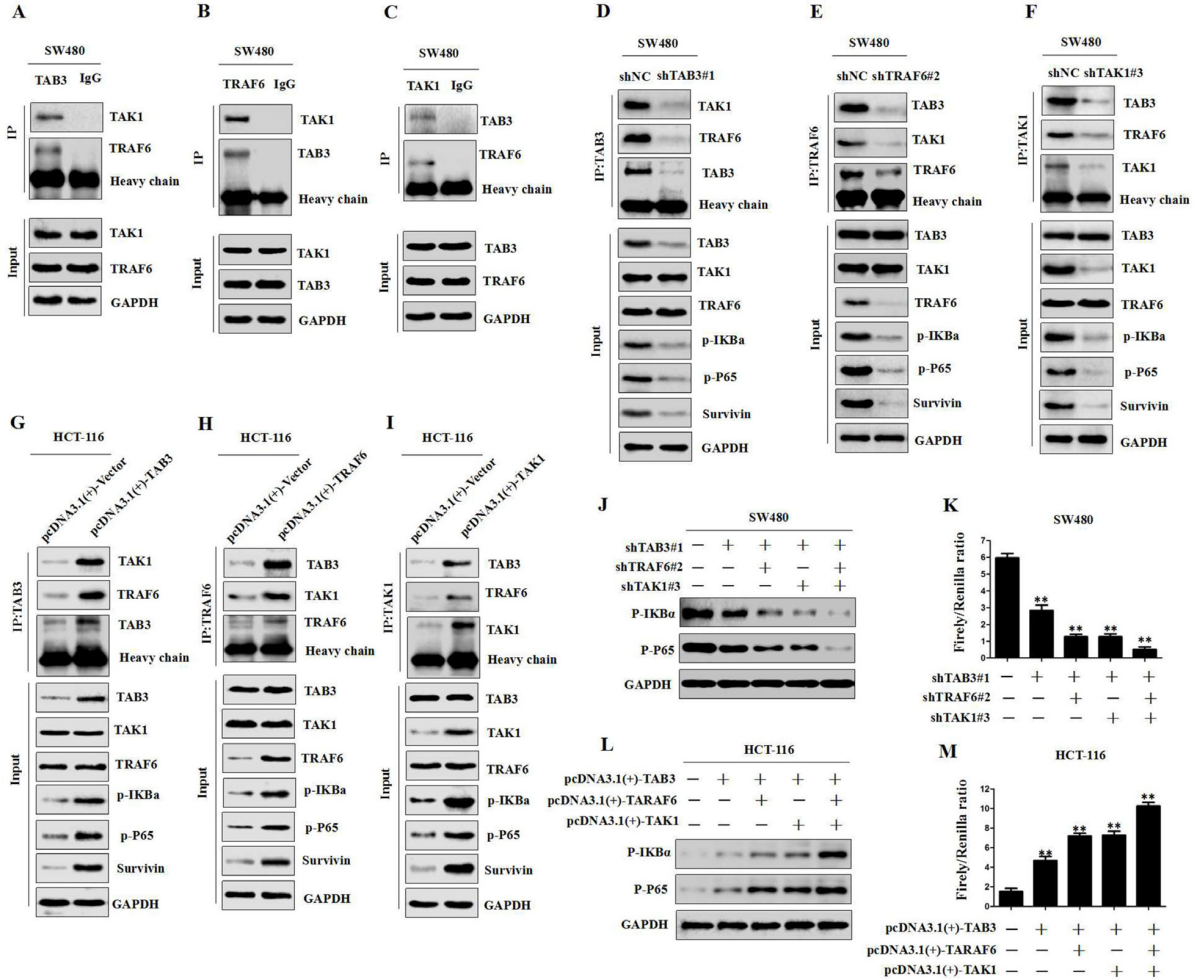
TAB3, TRAF6 and TAK1 are involved in NF-κB activation (**A**–**C**) co-IP among endogenous TAB3, TAK1 and TRAF6 in SW480 cells. (**D**–**I)** co-IP and Western blot analysis showing the levels of TAB3, TAK1 and TRAF6 after silencing or overexpression and their effects on TAB3, TAK1 and TRAF6 interaction and the NF-κB pathway in CRC cells. (**J–K**) Western blot and Luciferase analysis showing the levels of TAB3, TAK1 and TRAF6 after silencing and their effects on p-IKBa and p-P65 expression and NF-κB activity in SW480 cells (^**^P < 0.01). (**L**–**M**) Western blot and Luciferase analysis showing the levels of TAB3, TAK1 and TRAF6 after overexpression and their effects on p-IKBa and p-P65 expression and NF-κB activity in HCT-116 cells (^**^P < 0.01).

Previous studies have shown that TAB2, TRAF6 and TAK1 are involved in NF-κB activation [28, 29]. To determine whether the TAK1-TAB3-TRAF6 complex could also activate the NF-κB pathway in CRC cells, we first observed whether TAB3, TRAF6 and TAK1 directly interacted in CRC cells. The Co-IP study showed that endogenous TAB3, TRAF6 and TAK1 were detected in the immunoprecipitant, demonstrating the interaction among TAB3, TRAF6 and TAK1 (Figure 5A–5C and Supplementary Figure 4A–4C).In addition, we transfected shTAB3, shTRAF6 or shTAK1 plasmids into SW480 and LOVO cells and evaluated the effects of variable TAB3, TRAF6 and TAK1 complex on the NF-κB pathway. The results showed that the knockdown of TAB3, TRAF6 or TAK1 decreased the interaction among TAB3, TRAF6 and TAK1, and significantly decreased p-IKBα and p-p65 levels (Figure 5D–5F and Supplementary Figure 4D–4F). Whereas, the overexpression of TAB3, TRAF6 or TAK1 increased the TAB3, TRAF6 and TAK1 complex, as well as p-IKBα and p-p65 levels (Figure 5G–5I and Supplementary Figure 4G–4I). These data demonstrate that the TAK1-TAB3-TRAF6 complex is involved in NF-kB activation.

To further determine whether TAB3 activates NF-κB through the formation of the TAK1-TAB3-TRAF6 complex, we measured the changes in p-IKBα and p-p65 levels and NF-κB transcriptional activity in the synchronous TAB3, TAK1 and TRAF6 knockdown SW480 and LOVO cells. The results showed that reducing TAB3, TAK1 or TRAF6 expression could significantly inhibit p-IKBα and p-p65 levels and NF-κB transcriptional activity. Synchronous knockdown of TAB3, TAK1 and TRAF6 showed the strongest suppression effect (Figure 5J–5K and Supplementary Figure 4J–4K). Next, we investigated the effect of TAB3, TAK1 and TRAF6 synchronous overexpression on the NF-κB pathway. The results showed that the overexpression of TAB3, TAK1 or TRAF6 increased p-IκBα and p-p65 levels and NF-κB transcriptional activity, and the synchronous overexpression of TAB3, TAK1 and TRAF6 showed the strongest enhancement effect (Figure 5L–5M and Supplementary Figure 4L–4M). These data demonstrate that TAB3 activates NF-κB through binding to the TAK1-TRAF6 complex.

## DISCUSSION

Colorectal cancer (CRC) is one of the most common malignant cancers, and approximately 50–60% of patients present with metastases at the initial diagnosis [30, 31]. Tumor metastasis is a complex, multi-stage process that is classically simplified as local invasion, intravasation, survival in circulation, extravasation and colonization [32, 33]. The overexpression of metastasis initiation genes and their targets often predicts a poor prognosis in various types of cancer. Understanding the molecular mechanisms underlying CRC metastasis is of crucial significance to the development of therapeutic strategies for advanced CRC patients.

Transforming growth factor-β-activated kinase 1 (TAK1)-binding protein 3 (TAB3) belongs to a family of TABs that play an essential role in immunity [20]. Recent emerging evidence has indicated that TAB3 also plays a crucial role in cancer development and chemoresistance. For instance, the previously study confirmed that TAB3 is a downstream effector of TGFβ pathway [18], and TGFβ pathway has been demonstrated to be involved in the chemoresistance of CRC [34, 35]. Another study found that TAB3 was specifically expressed in triple-negative breast cancer, and it was significantly correlated with a poor prognosis in the patients [27]. However, the impact of TAB3 on CRC invasion and metastasis remains unclear.

In this study, we observed that TAB3 expression was significantly upregulated in CRC tissues and was associated with a poor prognosis in CRC patients. We also demonstrated that TAB3 promoted CRC cell invasion and metastasis both *in vitro* and *in vivo* and investigated the mechanism by which TAB3 affects invasion and metastasis in CRC cells.

Survivin, a member of the inhibitor of apoptosis family proteins [3], is highly expressed in CRC and associated with a poor clinical outcome [36]. In this study, we identified TAB3 as a novel regulator of Survivin. Our data indicate that TAB3 inhibition can reduce Survivin expression and decrease the invasion and metastasis of CRC *in vitro* and *in vivo*. Moreover, the upregulation of Survivin rescued the decreased invasion and liver metastasis induced by TAB3 knockdown. Whereas, inhibition of Survivin significantly decreased TAB3-induced invasiveness and metastasis. These results suggest that one of the mechanisms by which TAB3 promotes CRC metastasis involves the upregulation of Survivin expression.

Furthermore, the mechanism by which TAB3 regulates Survivin was further investigated. Our investigation focused on the NF-κB signaling pathway. We determined that the knockdown of TAB3 decreased p-IκBα and p-p65 levels and NF-κB activity. This ultimately lead to the downregulation of its target genes, such as c-Myc and MMP-9, whereas activation of NF-κB signaling rescued the decreased expression of Survivin and other NF-κB signaling components induced by TAB3 knockdown. Furthermore, the transwell assay indicated that the activation of NF-κB signaling significantly rescued the decreased cell migration and invasion induced by the knockdown of TAB3. Taken together, these studies demonstrated that TAB3 regulates Survivin-mediated CRC migration and invasion through the NF-κB pathway.

In addition, we further investigated the mechanism by which TAB3 regulates the NF-κB pathway. Previous studies confirmed that TAB2 acts as an adaptor that links TAK1 and TRAF6 to mediate NF-κB [28]. Comparative bioinformatics revealed that TAB3 is similar to TAB2 in that they both contain 3 putative functional domains with the same relative organization [29]. Such striking similarities lead us to hypothesize that the TAK1-TAB3-TRAF6 complex could also activate the NF-κB pathway in CRC cells. In this study, we demonstrated that TAB3, TRAF6 and TAK1 directly interacted in CRC cells and found that the TAB3, TRAF6 and TAK1 complex are involved in NF-κB activation in CRC cells. We also found that TAB3 activates NF-κB through the formation of the TAK1-TAB3-TRAF6 complex.

In summary, we demonstrated that TAB3 promotes CRC metastasis by regulating Survivin expression through the NF-κB signaling pathway by binding to the TAK1-TRAF6 complex. The newly identified TAB3–NF-κB–Survivin axis provides new insight into the underlying mechanisms of CRC invasion and metastasis, evidence of a valuable prognostic biomarker in CRC and a rationale for treatment of metastatic CRC.

## MATERIALS AND METHODS

### Patients and samples

Human CRC specimens were collected from 162 patients who underwent CRC resection at the Second Affiliated Hospital of Nanchang University and Jiangxi Provincial Cancer Hospital between January 2008 and December 2011. The study protocol was approved by the Ethics Committee of the Second Affiliated Hospital of Nanchang University and Jiangxi Provincial Cancer Hospital.

### Cell culture, plasmids and reagents

The human CRC cell lines SW480, SW620, LOVO, DLD-1 and HCT-116 were purchased from the Shanghai Institute of Cell Biology. The cells were cultured in RPMI 1640 medium (Gibco, Grand Island, NY, USA) supplemented with fetal bovine serum (HyClone, Logan, UT, USA) to a final concentration of 10% and antibiotics at 37°C with 5% CO_2_. Plasmids and reagents are described in the Supplementary Materials and Methods. All these cells were authenticated using short tandem repeat profiling by the Cell Bank.

### Immunohistochemistry

The CRC and adjacent tissues were treated with xylene, graded alcohol and then antigen retrieval was performed in 0.01M citrate buffer. Hydrogen peroxide was used for blockage. Tissue sections were treated with goat serum for 30 minutes. After that, the slides was incubated anti-TAB3 polyclonal antibodies (Abcam, Cambridge, MA, USA,1:300 dilution) overnight at 4°C. A 2-step immunohistochemical method (catalog:PV-9000; ZSGB-BIO Co., Ltd., Beijing, China) was adopted to perform the immunostaining. The staining intensity and percentage of positive cells were scored semi-quantitatively by 3 pathologists who were blind to the clinical parameters.

### qRT-PCR, H&E, western blot and co-immunoprecipitation (Co-IP) analyses

All qRT-PCR, western blot, and Co-IP procedures were performed as previously described [37]. The specific primers used for PCR amplification are shown in Supplementary Table 3.

### Wound-healing assay

The cells were grown to 80–90% confluence in 60 mm dishes. Artificial wounds were generated by scraping a pipette tip across the cell surface. After the removal of the detached cells by gentle washing with PBS, the cells were fed with fresh complete medium and were incubated over time to allow the cells to migrate into the open area. Cell movement during wound closure was measured by phase-contrast photography at 37°C for incubations of 0, 24 and 36 hrs, and 3 randomly selected wound areas were analyzed.

### Cell migration and invasion assay

For the migration assay, 5 × 10^4^ cells were resuspended in serum-free medium and were placed in the upper chambers. For the invasion assays, 1 × 10^5^ cells were seeded in a Matrigel-coated chamber (BD Biosciences, Bedford, MA, USA). After 24 hrs (to examine migration) or 48 hrs (to examine invasion) of incubation, the non-migrated cells on the upper surface of the membrane were removed, and the cells on the lower surface were fixed and stained with 0.1% crystal violet. The cells in 5 random microscopic fields were counted and imaged using a light microscope with a DP70 CCD system (Olympus Corp., Tokyo, Japan).

### Luciferase reporter assay

For the NF-κB transcriptional activity assay, 500 ng of PGL3.0-NF-ĸB-luc and 50 ng of Renilla luciferase plasmids or 500 ng of PGL3.0-luc and 50 ng of Renilla luciferase plasmids were co-transfected into TAB3-overexpression or TAB3-knockdown CRC cells. After 48 hrs of treatment, Luciferase and Renilla signals were measured using a Dual Luciferase Reporter Assay kit (Promega) according to the manufacturer’s protocol.

### *In vivo* metastasis assay

For *in vivo* metastasis assays, 1 × 10^7^ cells in 100 ml phosphate-buffered saline were inoculated subcutaneously onto the dorsal surfaces of BALB/c nude male mice (Shanghai SLAC Laboratory Animal Co., Ltd., China). Once xenografts were established, they were excised and minced into 1 mm^3^ pieces. One of these pieces was then orthotopically implanted at the ileocecal junction of another BALB/c nude mouse (eight in each group, male BALB/c-nu/nu, 6–8 weeks). The mice were sacrificed 35 days after tumor implantation [38]. The animal study was approved by the Ethics Committee for Animal Experiments of the Second Affiliated Hospital of Nanchang University and was performed in accordance with the“Guide for the Care and Use of Laboratory Animals” (revised 1985).

### Statistical analysis

All data were analyzed using SPSS (Statistical Package for the Social Sciences) 19.0 (SPSS, Inc.). The patient survival curve was calculated using the Kaplan-Meier method. Univariate and multivariate Cox proportional hazards regression analyses were performed to estimate the crude hazard ratios (HRs), adjusted HRs and 95% confidence intervals (CIs) of HRs. Differences between groups were analyzed using Student’s *t*-test when comparing two groups or one-way analysis of variance (ANOVA) when comparing more than 2 groups. Differences were considered statistically significant at *P <* 0.05.

## SUPPLEMENTARY MATERIALS FIGURES AND TABLES



## Abbreviations

TAK1: Transforming growth factor-β-activated kinase 1
TABs: TAK1-binding proteins
TRAFs: TNF-receptor-associated factors
CRC: colorectal cancer
IHC: immunohistochemistry
qRT-PCR: real-time quantitative polymerase chain reaction
Co-IP: co-immunoprecipitation
NSCLC: non-small cell lung cancer
