# Supplementary material for: TAB3 upregulates Survivin expression to promote colorectal cancer invasion and metastasis by binding to the TAK1-TRAF6 complex
Source: Oncotarget. 2017 Nov 18;8(63):106565–76. doi: 10.18632/oncotarget.22497 (PMC5739756; doi:10.18632/oncotarget.22497)
Supplement: Supplementary file 1 [file oncotarget-08-106565-s001.pdf]

TAB3 upregulates Survivin expression to promote colorectal cancer invasion and metastasis by binding to the TAK1-TRAF6 complex

SUPPLEMENTARY MATERIALS

Plasmids and reagents

Based on the TAB3 and Survivin sequences, 3 short hairpin RNAs (shRNAs) were designed using the siRNA Target Finder (InvivoGen). The target sites of shRNA are detailed in Supplementary Table 3. The shRNA sequences were cloned to pSIREN-RetroQ-TetH vector. The interference effects were confirmed by real-time quantitative polymerase chain reaction (qRT-PCR) and western blotting (Supplementary Figure 5).

The shTAB3, shSurvivin, shTAK1 and shTRAF6 vectors that produced the most significant knockdown effect were used to transduce CRC cells. In the stable transfection, the shTAB3 of CRC cells was selected based on resistance to hygromycin (600 μg/ml) (Invitrogen, Carlsbad, CA, USA), and CRC cells transfected with a negative control vector (shNC) were included as a control. The pcDNA3.1(+)-TAB3-expressing CRC cells were selected using G418 (700 μg/ml) (Invitrogen, Carlsbad, CA, USA), and an empty vector was used as the negative control. After 4 weeks of selection, individual colonies were isolated and expanded.

PGL3.0-NF-κB-luc, PGL3.0-luc and Renilla luciferase plasmids were purchased from Promega Corporation (Madison, WI, USA). pcDNA3.1(+)-TAB3,pcDNA3.1(+)-TAK1,pcDNA3.1(+)-TRAF6 and pcDNA3.1(+)-Survivin were constructed in our laboratory as previously described [1]. All the primers are shown in Supplementary Table 3.

The following antibodies and reagents were used: TAB3, Survivin, IKBa, p-IKBa, P65, p-P65, TAK1 and TRAF6 (Proteintech, Chicago, IL, USA), c-Myc and MMP-9 (Santa Cruz, CA, USA); protein A/G PLUS-agarose (Santa Cruz, CA, USA); Lipofectamine® 3000 (Invitrogen, Carlsbad, CA, USA); a total protein extraction kit (Applygen, Beijing, China); a BCA protein quantitation kit (Beyotime, Jiangsu, China); tumor necrosis factor (TNFa) and Caffeic Acid Phenethyl Ester (CAPE) (Sigma-Aldrich, St. Louis, MO, USA).

REFERENCES

1. Yuan R, Wang K, Hu J, Yan C, Li M, Yu X, Liu X, Lei J, Guo W, Wu L, Hong K, Shao J. Ubiquitin-like protein FAT10 promotes the invasion and metastasis of hepatocellular carcinoma by modifying β-catenin degradation. Cancer Res. 2014; 74:5287–300.

Supplementary Table 1: Univariate and multivariate analyses of overall survival in CRC patients (Cox proportional hazards regression model)

Parameters	Univariate analysis			Multivariate analysis		
	HR	95% CI	*P* value	HR	95% CI	*P* value
**Age (≥60 vs <60)**	1.300	0.839–2.014	0.240	—	—	—
**Gender (Male vs Female)**	1.184	0.768–1.826	0.445	—	—	—
**Tumor size (cm) (≥5 vs <5)**	1.266	0.823–1.947	0.283	—	—	—
**Histology (Undifferentiate vs Differentiated)**	1.384	0.829–2.311	0.213	—	—	—
**Lymphatic metastasis (Positive vs Negative)**	2.972	1.915–4.612	<0.001**	2.136	1.322–3.453	0.002**
**Venous invasion (Positive vs Negative)**	2.693	1.711–4.238	<0.001**	1.749	1.070–2.860	0.026**
**TNM staging (III/IV vs I/II)**	2.304	1.487–3.569	<0.001**	1.729	1.095–2.732	0.019**
**TAB3 expression (High vs Low)**	2.348	1.496–3.685	<0.001**	1.657	1.035–2.652	0.035**

$P < 0.05$ was considered as statistically significant.

Supplementary Table 2: Univariate and multivariate analyses of disease-free survival in CRC patients (Cox proportional hazards regression model)

Parameters	Univariate analysis			Multivariate analysis		
	HR	95% CI	*P* value	HR	95% CI	*P* value
**Age (≥60 vs <60)**	1.127	0.753–1.686	0.563	—	—	—
**Gender(Male vs Female)**	1.128	0.758–1.678	0.551	—	—	—
**Tumor size (≥5 vs <5)**	1.132	0.763–1.679	0.539	—	—	—
**Histology (Undifferentiated vs Differentiated)**	1.374	0.840–2.247	0.205	—	—	—
**Lymphatic metastasis (Positive vs Negative)**	4.145	2.732–6.289	<0.001**	3.557	2.312–5.472	<0.001**
**Venous invasion (Positive vs Negative)**	2.231	1.481–3.361	<0.001**	2.029	1.306–3.153	0.002**
**TNM staging (III/IV vs I/II)**	2.169	1.453–3.238	<0.001**	1.844	1.190–2.859	0.006**
**TAB3 expression (High vs Low)**	2.274	1.506–3.434	<0.001**	1.637	1.058–2.533	0.027**

$P < 0.05$ was considered as statistically significant.

Supplementary Table 3: Primers and shRNA target sequences

Name	Sequences	Enzyme
Primers for real-time PCR:		
TAB3 sense:	5′-CCTGAAATTCCAGAGGGCGT-3′	
TAB3 antisense:	5′-TGCTGCATGCTGAGGATCAA -3′	
Survivin sense:	5′-TGACGACCCCATAGAGGAACA-3′	
Survivin antisense:	5′- CGCACTTTCTCCGCAGTTTC-3′	
GAPDH sence:	5′-CTATAAATTGAGCCCGCAGCC-3′	
GAPDH antisence:	5′-ACTGTGGTCATGAGTCCTTCC-3′	
Primers for plasmids construct:		
pcDNA3.1(+)-TAB3-sence:	5′- GGAGACCCAAGCTGGCTAGCATGTACCCTTATGAT GTCCCAGACTATGCTATGGCGCAAAGCAGCCCACA-3′	NheI
pcDNA3.1(+)-TAB3-antisence:	5′-AGTCCAGTGTGGTGGAATTCTCAGGTGTACCGTGG CATCTCGCAC-3′	EcoRI
pcDNA3.1(+)-Survivin- sence:	5′-ACGGGCCCTCTAGACTCGAGCGCCACCATGGGTGC CCCGACGTTGCC-3′	XhoI
pcDNA3.1(+)-Survivin- antisence:	5′-AGTCCAGTGTGGTGGAATTCATCCATGGCAGCCAG CTGCTCG-3′	EcoRI
pcDNA3.1(+)-TRAF6-sence:	5′- GTCCGGACTCAGATCTCGAGCTATGAGTCTGCTAA ACTGTGAAAACAG-3′	XhoI
pcDNA3.1(+)-TRAF6-antisence:	5′-TATCTAGATCCGGTGGATCCCTATACCCCTGCATCA GTACTTCGTG-3′	BamHI
pcDNA3.1(+)-TAK1- sence:	5′-ACGGGCCCTCTAGACTCGAGATGCATCATCATCATC ATCATATGTCTACAGCCTCTGCC-3′	XhoI
pcDNA3.1(+)-TAK1- antisence:	5′-TTAAACTTAAGCTTGGTACCTCATGAAGTGCCTTGT CGTTTCTGC-3′	KpnI
The target sites of shRNA:		
shTAB3#1	5′-GGTTGAAGTCTGAAGTTAA-3′	
shTAB3#2	5′-TCCTTCATACATGCACATA-3′	
shTAB3#3	5′-CAACTTAATGGTGGTCGAA-3′	
shSurvivin#1	5′-GCATCTCTACATTCAAGAA-3′	
shSurvivin#2	5′-CCAACAATAAGAAGAAAGA-3′	
shSurvivin#3	5′-CCACTGAGAACGAGCCAGA-3′	
shTAK1#1	5′-CGCAAUGAGUUGGUGUUUATT-3′	
shTAK1#2	5′-CGGAAACCCUUUGAUGAGATT-3′	
shTAK1#3	5′-GGCAAAGCAACAGAGUGAATT-3′	
shTRAF6#1	5′-GGGUACAAUACGCCUUACATT-3′	
shTRAF6#2	5′-GCAGUGCAAUGGAAUUUAUTT-3′	
shTRAF6#3	5′-GAUCCAGGGAUAUGAUGUATT-3′	
shNC	5′-TTCTCCGAACGTGTCACGT-3′	

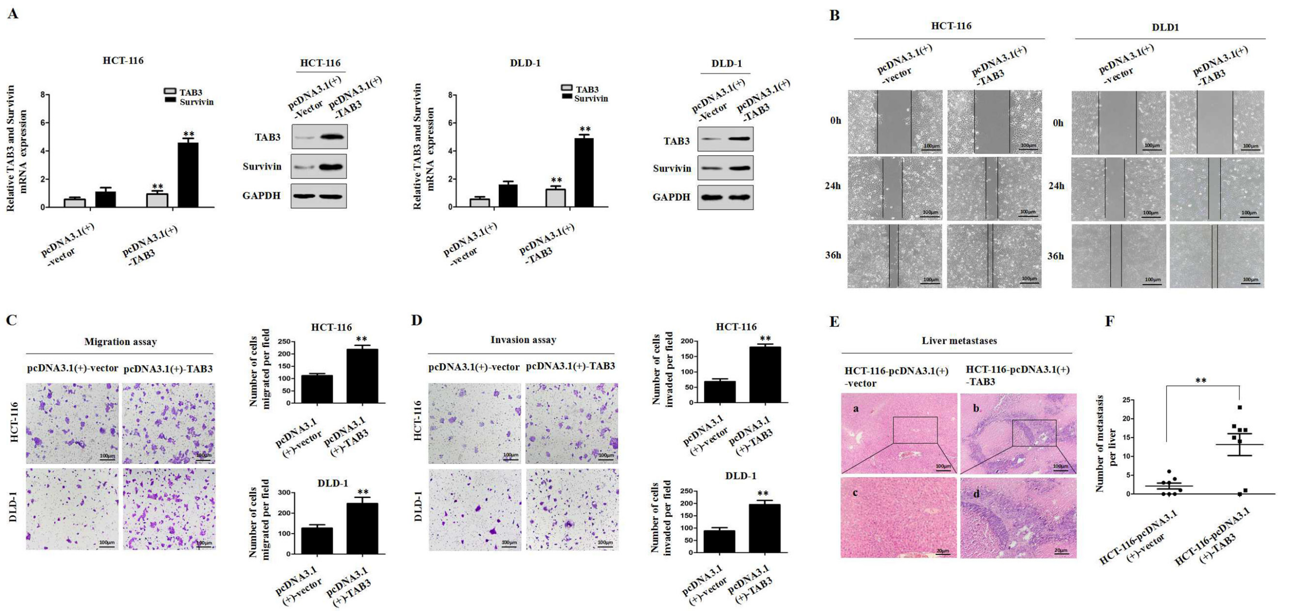

Supplementary Figure 1: Overexpression of TAB3 increased Survivin expression and enhanced CRC invasion and metastasis *in vitro* and *in vivo*. (**A**) Western blot analyses were used to detect TAB3 and Survivin expression in HCT-116 and DLD-1 cells stably transfected with pcDNA3.1(+)-Vector and pcDNA3.1(+)-TAB3. (**B**) Wound healing assay. Wound closure was delayed in stable TAB3-overexpression cells compared with the pcDNA3.1(+)-Vector control at both the 24- and 36-h time points. Magnification, ×100. (**C**–**D**) Transwell migration and invasion assays of HCT-116 and DLD-1 cells with TAB3 overexpression ($^{*}P < 0.05$). Magnification, ×100. (**E**) Representative H&E staining of livers from the HCT-116-pcDNA3.1(+)-Vector and HCT-116-pcDNA3.1(+)-TAB3 groups. Magnification: a and b, ×100; c and d, ×400. (**F**) ($n = 8$; $^{**}P < 0.01$).

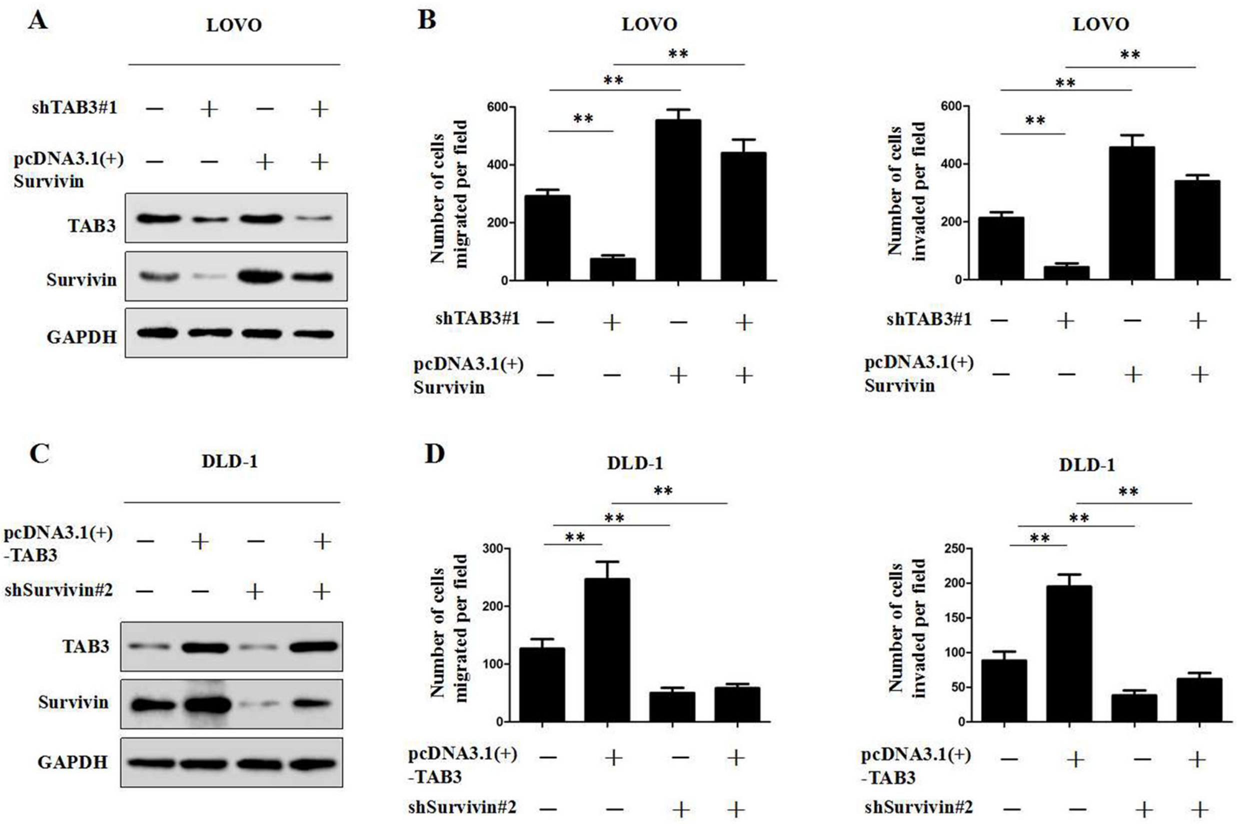

Supplementary Figure 2: TAB3 promotes CRC migration and invasion by upregulating Survivin expression. (**A**) The upregulation of Survivin attenuated the loss of Survivin expression in LOVO-shTAB3 cells. (**B**) Transwell assays showed that the upregulation of Survivin significantly rescued the cell migration and invasion in LOVO-shTAB3 cells ($^{**}P < 0.01$). (**C**) Protein levels of TAB3 and Survivin were detected by western blot analysis. The knockdown of Survivin expression dramatically inhibited the increase of Survivin expression in DLD-1-TAB3 cells. (**D**) Transwell assays showed that Survivin inhibition reduced TAB3-enhanced cell migration and invasion ($^{**}P < 0.01$).

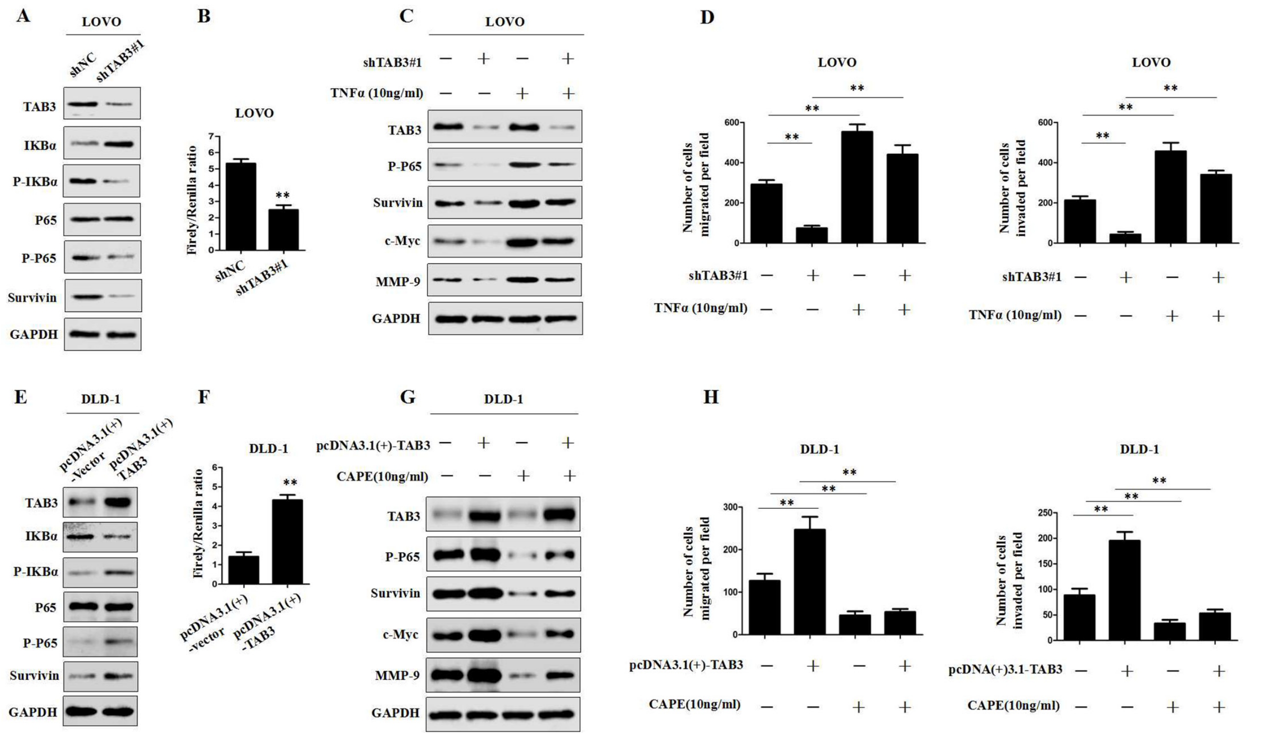

Supplementary Figure 3: TAB3 regulates Survivin expression through the NF-κB pathway. (**A**) The protein expression levels of TAB3, IKBa, p-IKBa, P65, p-P65 and Survivin were assessed by Western blotting in TAB3-silenced LOVO cells. (**B**) Luciferase analysis was used to determine the NF-κB activity in TAB3-knockdown LOVO cells ($^{**}P < 0.01$). (**C**) Western blot analysis showing the levels of TAB3 silencing and activation of the NF-κB pathway and their effects on p-P65, Survivin, c-Myc, and MMP-9 in LOVO cells. (**D**) Activation of the NF-κB pathway rescued the cell migration and invasion in LOVO-shTAB3 cells ($^{**}P < 0.01$). (**E**) Protein expression levels of TAB3, IKBa, p-IKBa, P65, p-P65 and Survivin in DLD-1 cells transfected with pcDNA3.1(+)-vector and pcDNA3.1(+)-TAB3. (**F**) Luciferase analysis was performed using DLD-1 cells transfected with the pcDNA3.1(+)-vector or pcDNA3.1(+)-TAB3 plasmid ($^{**}P < 0.01$). (**G**) Western blot analysis showing the levels of TAB3 overexpression and NF-κB signaling inhibition (treatment with 10 ng/ml Caffeic Acid Phenethyl Ester) and their effects on p-P65, Survivin, c-Myc and MMP-9 in DLD-1 cells. (**H**) Blockade of the NF-κB pathway enhanced migration and invasion in DLD-1-pcDNA3.1(+)-TAB3 cells ($^{**}P < 0.01$).

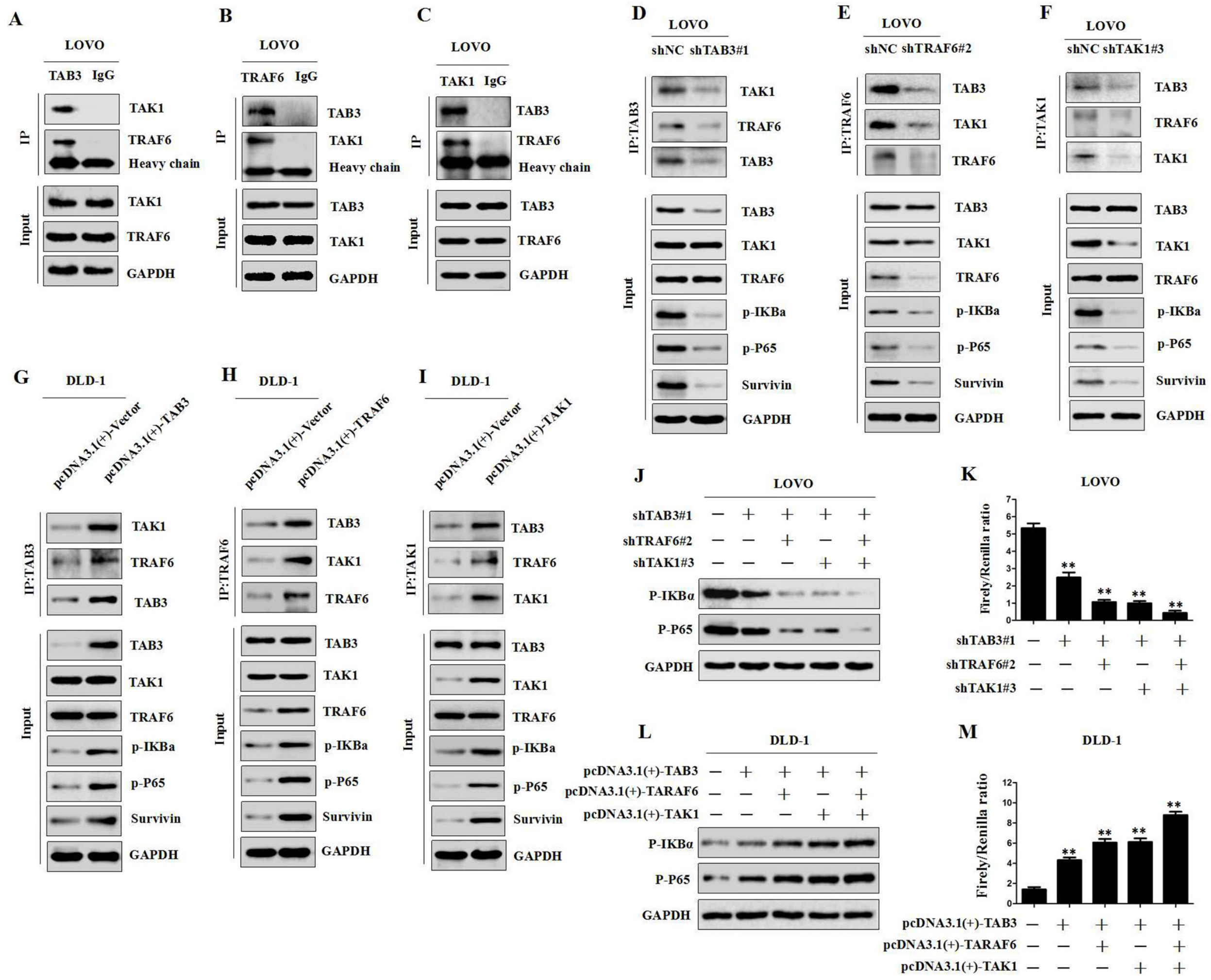

Supplementary Figure 4: TAB3, TRAF6 and TAK1 are involved in NF-κB activation. (**A**–**C**) co-IP among endogenous TAB3, TAK1 and TRAF6 in LOVO cells. (**D**–**I**) co-IP and western blot analysis showing the levels of TAB3, TAK1 and TRAF6 after silencing or overexpression and their effects on TAB3, TAK1 and TRAF6 interaction and the NF-κB pathway in CRC cells. (**J**–**K**) Western blot and Luciferase analysis showing the levels of TAB3, TAK1 and TRAF6 after silencing and their effects on p-IKBa and p-P65 expression and NF-κB activity in LOVO cells ($^{**}P < 0.01$). (**L**–**M**) Western blot and Luciferase analysis showing the levels of TAB3, TAK1 and TRAF6 after overexpression and their effects on p-IKBa and p-P65 expression and NF-κB activity in DLD-1 cells ($^{**}P < 0.01$).

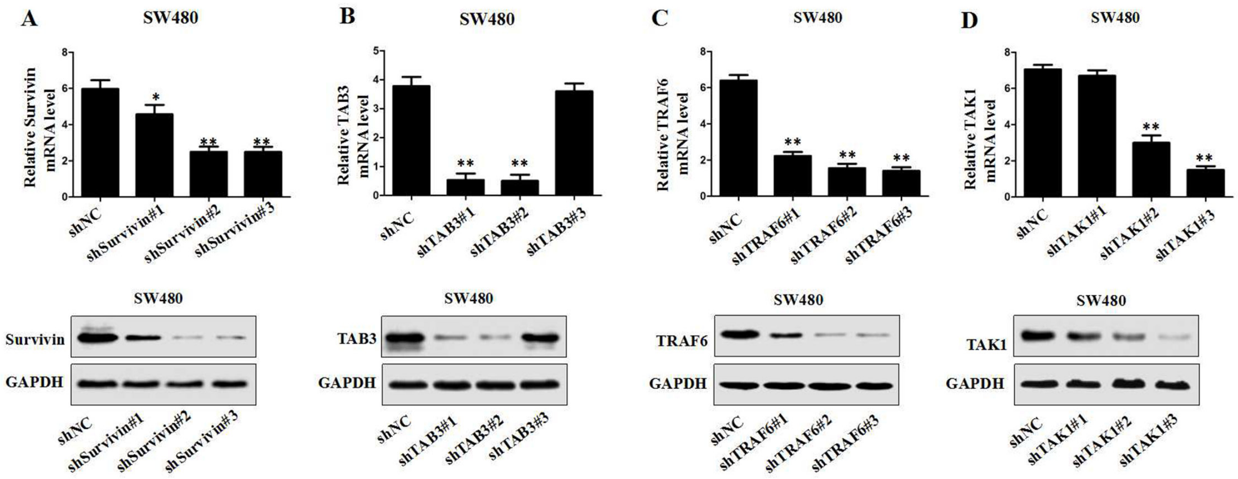

Supplementary Figure 5: The TAB3, TAK1, TRAF6 and Survivin expression levels were decreased after transfection of the corresponding shRNA plasmids. (**A–D**) The changes in TAB3, TAK1, TRAF6 and Survivin expression levels were evaluated by qRT-PCR and Western blot analysis ($^{**}p < 0.01$).
